# Validation and bioinformatics analysis of differentially expressed circRNAs involved in developing male *Xenopus laevis* chronically exposed to atrazine

**DOI:** 10.1016/j.dib.2018.04.011

**Published:** 2018-04-10

**Authors:** Linlin Sai, Ling Li, Chenyang Hu, Binpeng Qu, Qiming Guo, Qiang Jia, Yu Zhang, Cunxiang Bo, Xiangxin Li, Hua Shao, Jack C. Ng, Cheng Peng

**Affiliations:** a Shandong Academy of Occupational Health and Occupational Medicine, Ji’nan, Shandong, China; b Shandong Medical College, Ji’nan, Shandong, China; c Heze Center for Disease Control and Prevention, Heze, Shandong, China; d The University of Queensland, Queensland Alliance for Environmental Health Sciences (QAEHS), Australia

**Keywords:** Herbicide, CircRNA, Q-RT-PCR, CircRNA-miRNA, KEGG, Reproductive toxicology

## Abstract

The data presented here are related to the research article titled “Identification of circular RNAs and their alterations involved in developing male *Xenopus laevis* chronically exposed to atrazine” (Sai et al., 2018) [1]. Circular RNAs (circRNAs) are implicated in multiple developmental anomalies (Bachmayr-Heyda et al., 2015; Li et al., 2015) [2], [3]. This report describes the differentially expressed circRNAs involved in developing male *Xenopus laevis (X. laevis)* chronically exposed to atrazine (AZ) database. The database contains the validation of differentially expressed circRNAs, KEGG analysis of differentially expressed circRNA-associated target genes and prediction of miRNA binding sites. These data may help to further evaluate the role of circRNAs in male *X. laevis* chronically exposed to AZ.

**Specifications Table**

**Table:** 

Subject area	*Environmental toxicology*
More specific subject area	*CircRNA study involved in developing male Xenopus laevis chronically exposed to 100 µg/L AZ*
Type of data	*Table*
How data was acquired	*Quantitative RT-PCR (ViiA 7 Real-time PCR System, Applied Biosystems, Carlsbad, CA, USA), Kyoto Encyclopedia of Genes and Genomes (KEGG), miRNA-binding sites on circRNAs are predicted by custom-written software based on Targetscan and Miranda software (Cloud-Seq Biotech Ltd. Co., Shanghai, China)*
Data format	*Analyzed*
Experimental factors	*cDNA amplification, RNA library construction, RNA-sequencing, PCR analysis, bioinformatics analysis*
Experimental features	*Validation of differentially expressed circRNAs by Q-RT-PCR, KEGG analysis of differentially expressed circRNA-associated target genes and prediction of miRNA binding sites*
Data source location	*Ji’nan, Shandong, China*
Data accessibility	*This is an innovative data, not yet published elsewhere*
Related research article	*Identification of circular RNAs and their alterations involved in developing male X. laevis chronically exposed to AZ.*

**Value of the data**

1. The explored data are innovative information.
2. Data represented are unequivocal and innovative research based work. Data would be valuable to the researcher those who are doing research on circRNAs in developing male *X. laevis* chronically exposed to AZ.
3. Data are valuable for estimating effects of the AZ on male amphibians.
4. Data will help to understand the normal function of circRNAs as well as their roles in response to environmental chemicals like AZ.

## 1 Data

The dataset of this article provides information on the differentially expressed circRNAs involved in developing male *Xenopus laevis* chronically exposed to AZ. Table 1 shows the sequences of primers in the Q-RT-PCR assay for validating the differential expressions of circRNAs involving in developing male *X. laevis* chronically exposed to 100 µg/L AZ after sequencing. In Table 2, data show the results of Q-RT-PCR validation of the differentially expressed circRNAs. KEGG analysis were performed for the differentially expressed circRNA-associated target genes [4], [5]. Table 3 shows the 19 enrichment pathways of differentially expressed circRNAs-associated target genes. MiRNA-binding sites on circRNAs predicted by custom-written software based on Targetscan and Miranda software (Cloud-Seq Biotech Ltd. Co., Shanghai, China). From the data (Table 4), it can be seen that 282 circRNAs linked to *X. laevis* exposed to 100 µg/L AZ for 180 days had predicted miRNA targets by custom-written software.

**Table 1:** Sequences of primers in the Q-RT-PCR assay for validating the differential expressions of circRNAs after sequencing.

Target name	Primer name	Sequence (5′→3′)	Product length
NC_030732.1:43721272–43728946-	1-Forward	GGCTATGGTAACCGTGGAAA	186
	1-Reverse	TTTTCACACCCCGTTTCTTC	
			
NC_030734.1:102788414–102788574-	2-Forward	AGCAAGAAATGTTGCTGGAGA	136
	2-Reverse	GTCCCTGTCCCTGAGAGGTT	
			
NC_030734.1:106398702–106449286+	3-Forward	CCTGTTGCTTAGGCCATCAT	213
	3-Reverse	TGTGATGAGGCAGAGTTTCG	
			
NC_030734.1:14617230–14633443-	4-Forward	CTAACACCACCCTCGTCCAT	193
	4-Reverse	TCATTCTGGTCAAGGGCTTT	
			
NC_030735.1:109227383–109290634-	5-Forward	ACTGCAGGCATCTCTTGGAC	200
	5-Reverse	GCCAAAAACAACTGCCAAAT	
			
NC_030737.1:31865134–31952348-	6-Forward	CATTTTGCATTGGCATGTTT	188
	6-Reverse	ATCGCACAAAGTCCCATCTC	
			
NC_030738.1:39776310–39778799-	7-Forward	TCAGAATGCAGCTGTCAAGG	199
	7-Reverse	CTGGATTTCCACCATCCTCA	
			
NC_030740.1:57091911–57092116+	8-Forward	GGCTACCAGAATCGTCATGC	197
	8-Reverse	ATGCTGTTGCTGATTGGATG	
			
NW_016694872.1:550852-632532-	9-Forward	CTTCTGTGGTCACCGGAAGT	189
	9-Reverse	TGGACATGTGAACTGGGTGT	
			
NC_030738.1:21126033–21131815+	10-Forward	CCATGCATTCCACTAGGTCA	191
	10-Reverse	ATCACTCACTGACGCACGAG	
			
NM_001092296.1	11-Forward	GAGGACCTCGTGTGTGGTTT	60
	11-Reverse	CCAGAGTTTTGGCAATGTGA

**Table 2:** The results of Q-RT-PCR validation of the differentially expressed circRNAs.

Differentially expressed circRNAs	2-△△ CT	*P*-value
NC_030732.1:43721272–43728946-	0.519	0.037
NC_030734.1:102788414–102788574-	0.457	0.046
NC_030734.1:106398702–106449286+	0.561	0.049
NC_030735.1:109227383–109290634-	0.490	0.047
NC_030737.1:31865134–31952348-	0.802	0.014
NC_030738.1:39776310–39778799-	2.480	0.040
NW_016694872.1:550852-632532-	0.472	0.045
NC_030738.1:21126033–21131815+	10.512	0.036

Sequences of the primers used were listed in Table 1.

**Table 3:** The enrichment pathways of differentially expressed circRNAs-associated target genes by KEGG.

PathwayID	Definition	Fisher-*P*value	Count	FDR	Enrichment Score	Gene Ratio
xla04310	Wnt signaling pathway	0.000625153	273	0.08756559	3.204014	0.06
xla04510	Focal adhesion	0.00104869	368	0.08756559	2.979353	0.0725
xla04114	Oocyte meiosis	0.001798677	196	0.1001263	2.745047	0.045
xla04010	MAPK signaling pathway	0.003075789	468	0.1284142	2.512044	0.0825
xla04622	RIG-I-like receptor signaling pathway	0.005122665	120	0.1523613	2.290504	0.03
xla04115	p53 signaling pathway	0.005474059	121	0.1523613	2.261691	0.03
xla04012	ErbB signaling pathway	0.006477662	155	0.1545385	2.188582	0.035
xla04340	Hedgehog signaling pathway	0.007444835	81	0.1554109	2.128145	0.0225
xla04920	Adipocytokine signaling pathway	0.01074467	132	0.1993733	1.968807	0.03
xla00480	Glutathione metabolism	0.01256994	88	0.2039134	1.900667	0.0225
xla04912	GnRH signaling pathway	0.01343142	186	0.2039134	1.871878	0.0375
xla00515	Mannose type O-glycan biosynthesis	0.01902007	50	0.2545346	1.720788	0.015
xla04914	Progesterone-mediated oocyte maturation	0.0212995	162	0.2545346	1.671631	0.0325
xla04146	Peroxisome	0.02316556	130	0.2545346	1.635157	0.0275
xla00601	Glycosphingolipid biosynthesis - lacto and neolacto series	0.02344076	82	0.2545346	1.630028	0.02
xla04144	Endocytosis	0.02438655	502	0.2545346	1.61285	0.0775
xla04068	FoxO signaling pathway	0.02877994	259	0.2827206	1.54091	0.045
xla04810	Regulation of actin cytoskeleton	0.03934001	384	0.3649879	1.405165	0.06
xla00533	Glycosaminoglycan biosynthesis - keratan sulfate	0.04531492	46	0.3982943	1.343759	0.0125

**Table 4:** The top 5 predicted miRNA targets for 282 circRNAs linked to *X. laevis* exposed to 100 µg/L AZ for 180 days.

circRNA	miRNA	miRNA	miRNA	miRNA	miRNA
NC_030728.1:107931245–107932206+	xla-miR-133d	xla-miR-133a	xla-miR-133b		
NC_030740.1:64855970–64877155+	xla-miR-18	xla-miR-19b			
NC_030741.1:10475414–10475723+	xla-miR-18				
NC_030726.1:160156875–160159679-	xla-miR-205				
NC_030734.1:106485869–106486720-	xla-miR-427	xla-miR-133d	xla-miR-133b	xla-miR-133a	
NC_030726.1:76691651–76698216+	xla-miR-15c	xla-miR-429	xla-miR-194	xla-miR-703	xla-miR-23a
NC_030724.1:25016701–25021875+	xla-miR-427	xla-miR-363	xla-miR-223	xla-miR-133a	xla-miR-133b
NC_030726.1:102820197–102823698-	xla-miR-92a	xla-miR-133a	xla-miR-133d	xla-miR-133b	
NC_030736.1:12689783–12690847-	xla-miR-19b				
NC_030727.1:94629493–94639781+	xla-miR-15c	xla-miR-19b	xla-miR-20	xla-miR-18	xla-miR-429
NC_030737.1:50553238–50553849-	xla-miR-427	xla-miR-428			
NC_030726.1:154989366–154998753+	xla-miR-703	xla-miR-427	xla-miR-205		
NC_030733.1:34483227–34511810-	xla-miR-15c	xla-miR-20	xla-miR-19b	xla-miR-1b	xla-miR-18
NC_030732.1:108698546–108701014-	xla-miR-23a	xla-miR-92a			
NC_030741.1:23757079–23761365+	xla-miR-223				
NW_016694888.1:355851-357777+	xla-miR-24b	xla-miR-1b	xla-miR-194	xla-miR-429	xla-miR-18
NC_030725.1:145886684–145893268-	xla-miR-427	xla-miR-363	xla-miR-428	xla-miR-23a	
NC_030725.1:160273437–160274678+	xla-miR-429				
NC_030736.1:93771653–93772342+	xla-miR-427	xla-miR-18	xla-miR-703	xla-miR-428	
NC_030725.1:33745008–33747282-	xla-miR-19b	xla-miR-223	xla-miR-194	xla-miR-24b	xla-miR-1b
NC_030736.1:95594264–95594855+	xla-miR-428	xla-miR-427	xla-miR-20		
NC_030729.1:59204043–59212585+	xla-miR-205	xla-miR-15c	xla-miR-1306		
NW_016694811.1:4160983-4163596-	xla-miR-15c	xla-miR-92a			
NC_030725.1:75897770–75940162-	xla-miR-15c				
NC_030732.1:53423582–53432267+	xla-miR-19b	xla-miR-15c	xla-miR-703		
NC_030734.1:85835741–85846351-	xla-miR-20	xla-miR-133d	xla-miR-427	xla-miR-142	xla-miR-15c
NC_030732.1:43721272–43728946-	xla-miR-19b	xla-miR-205	xla-miR-133d	xla-miR-133b	xla-miR-133a
NC_030732.1:22358736–22365805-	xla-miR-205	xla-miR-363	xla-miR-18	xla-miR-19b	
NC_030741.1:63398525–63424921-	xla-miR-429				
NC_030726.1:153762674–153762866-	xla-miR-20	xla-miR-427	xla-miR-428		
NC_030727.1:94976453–94990031-	xla-miR-142				
NC_030740.1:14329209–14332656+	xla-miR-15c	xla-miR-24b			
NC_030726.1:68684168–68687145+	xla-miR-19b				
NC_030728.1:62905308–62912700-	xla-miR-15c				
NC_030735.1:109227383–109290634-	xla-miR-18	xla-miR-15c	xla-miR-427	xla-miR-20	xla-miR-223
NC_030737.1:80523685–80526513+	xla-miR-429				
NC_030734.1:94791370–94802295-	xla-miR-142				
NC_030731.1:70450630–70453977-	xla-miR-15c	xla-miR-1b			
NW_016694850.1:165938-168684-	xla-miR-19b	xla-miR-23a	xla-miR-24b		
NC_030724.1:162927448–162932292+	xla-miR-205	xla-miR-23a	xla-miR-703		
circRNA	miRNA	miRNA	miRNA	miRNA	miRNA
NC_030726.1:170141772–170152857+	xla-miR-19b	xla-miR-363	xla-miR-133d	xla-miR-133b	xla-miR-133a
NC_030724.1:20602441–20604471-	xla-miR-205				
NC_030732.1:93793150–93820672+	xla-miR-1b				
NC_030728.1:130780434–130781838-	xla-miR-23a	xla-miR-19b			
NC_030730.1:87897066–87916110-	xla-miR-427				
NC_030724.1:16657286–16660198+	xla-miR-15c	xla-miR-1306			
NC_030737.1:38242321–38257632+	xla-miR-23a	xla-miR-142			
NC_030731.1:109099315–109104730-	xla-miR-19b	xla-miR-223	xla-miR-427	xla-miR-1b	xla-miR-20
NC_030725.1:12393643–12395967-	xla-miR-23a				
NC_030726.1:27655736–27658064+	xla-miR-223	xla-miR-1b			
NC_030727.1:67838131–67839236-	xla-miR-223	xla-miR-18			
NC_030740.1:78837348–78837807-	xla-miR-1b				
NC_030739.1:3829594–3830014+	xla-miR-205	xla-miR-19b			
NC_030724.1:174501216–174522171+	xla-miR-19b				
NC_030725.1:115717672–115718776-	xla-miR-18	xla-miR-20	xla-miR-429	xla-miR-133d	xla-miR-1b
NC_030734.1:41157487–41160006+	xla-miR-205	xla-miR-20			
NC_030732.1:19072979–19073580-	xla-miR-428	xla-miR-427			
NC_030734.1:109280643–109285081-	xla-miR-194				
NC_030733.1:50631948–50642076-	xla-miR-427	xla-miR-428	xla-miR-703		
NC_030726.1:130247524–130272922+	xla-miR-15c	xla-miR-194			
NC_030731.1:80596587–80599136-	xla-miR-142	xla-miR-23a	xla-miR-427	xla-miR-20	xla-miR-428
NC_030739.1:36191184–36191805+	xla-miR-20	xla-miR-19b			
NC_030725.1:11616097–11616889+	xla-miR-1306				
NC_030727.1:128265094–128266804+	xla-miR-427	xla-miR-24b	xla-miR-703		
NC_030740.1:105999689–106002961-	xla-miR-19b				
NC_030733.1:23052893–23053431+	xla-miR-703				
NC_030730.1:70393563–70405777+	xla-miR-20	xla-miR-1b	xla-miR-142	xla-miR-428	xla-miR-223
NC_030737.1:83574596–83583489+	xla-miR-15c	xla-miR-427	xla-miR-428	xla-miR-194	xla-miR-18
NC_030727.1:66053235–66057876-	xla-miR-1b	xla-miR-20	xla-miR-428	xla-miR-427	
NC_030728.1:62937674–62947488+	xla-miR-703	xla-miR-18			
NC_030738.1:68976380–68977086-	xla-miR-18	xla-miR-427			
NW_016694892.1:26153-27290+	xla-miR-363				
NC_030724.1:143151804–143152935-	xla-miR-18				
NC_030734.1:98881558–98914095-	xla-miR-23a	xla-miR-19b			
NC_030734.1:57200126–57207190+	xla-miR-1b				
NC_030732.1:62658175–62664193+	xla-miR-205	xla-miR-429			
NC_030729.1:59341935–59363703-	xla-miR-427	xla-miR-18	xla-miR-15c	xla-miR-703	xla-miR-429
NC_030740.1:66890501–66890846+	xla-miR-142	xla-miR-15c			
NC_030738.1:55306347–55307113+	xla-miR-20	xla-miR-363			
NC_030732.1:49586121–49588850-	xla-miR-205	xla-miR-19b			
NC_030741.1:101502504–101503105+	xla-miR-15c				
NW_016694865.1:628498-632223+	xla-miR-23a				
circRNA	miRNA	miRNA	miRNA	miRNA	miRNA
NC_030724.1:35396202–35400617+	xla-miR-23a				
NC_030733.1:6201642–6201910+	xla-miR-223	xla-miR-24b	xla-miR-205		
NC_030735.1:17768662–17785569+	xla-miR-23a				
NC_030727.1:132699431–132699787-	xla-miR-15c	xla-miR-428			
NC_030724.1:105611901–105637401+	xla-miR-15c	xla-miR-142	xla-miR-427	xla-miR-223	xla-miR-18
NC_030729.1:64339785–64361328+	xla-miR-427	xla-miR-133a	xla-miR-133d	xla-miR-133b	xla-miR-15c
NC_030737.1:32708865–32717748-	xla-miR-428	xla-miR-20			
NC_030732.1:65055266–65057014-	xla-miR-15c				
NC_030735.1:95974107–95989476-	xla-miR-92a	xla-miR-703	xla-miR-1b	xla-miR-205	
NC_030727.1:77776018–77777622+	xla-miR-19b	xla-miR-20	xla-miR-142	xla-miR-427	
NC_030738.1:1661483–1662700-	xla-miR-19b	xla-miR-15c	xla-miR-133a	xla-miR-133d	xla-miR-133b
NC_030728.1:123007127–123007546-	xla-miR-703				
NC_030739.1:7513676–7514449-	xla-miR-133d	xla-miR-133b	xla-miR-133a		
NC_030740.1:75628520–75629574-	xla-miR-1b	xla-miR-429	xla-miR-428	xla-miR-427	xla-miR-23a
NC_030732.1:74604196–74606604+	xla-miR-19b	xla-miR-15c	xla-miR-142	xla-miR-194	xla-miR-133d
NC_030730.1:10150206–10153200+	xla-miR-18				
NC_030735.1:8450716–8468533-	xla-miR-20	xla-miR-142			
NC_030729.1:70852510–70871306+	xla-miR-142				
NC_030732.1:1244473–1252348+	xla-miR-429	xla-miR-18	xla-miR-703	xla-miR-15c	xla-miR-142
NC_030728.1:107378386–107381277+	xla-miR-23a	xla-miR-133a	xla-miR-703		
NC_030724.1:179432037–179435517-	xla-miR-18	xla-miR-1b			
NC_030734.1:106398702–106449286+	xla-miR-18	xla-miR-20	xla-miR-142	xla-miR-223	xla-miR-15c
NC_030729.1:64054605–64065892-	xla-miR-20	xla-miR-428	xla-miR-15c	xla-miR-223	xla-miR-92a
NC_030724.1:170644099–170649196+	xla-miR-427	xla-miR-428	xla-miR-205	xla-miR-19b	
NC_030729.1:37773578–37779829-	xla-miR-24b				
NC_030731.1:51165922–51175381-	xla-miR-1306				
NC_030724.1:175775086–175790852+	xla-miR-133d	xla-miR-133b	xla-miR-133a	xla-miR-19b	xla-miR-23a
NC_030728.1:90621739–90639104+	xla-miR-133d	xla-miR-133b	xla-miR-133a	xla-miR-20	xla-miR-18
NC_030734.1:74355300–74391682-	xla-miR-427	xla-miR-19b	xla-miR-15c		
NC_030741.1:25327114–25329691-	xla-miR-223	xla-miR-1b	xla-miR-20		
NC_030735.1:35939491–35949048+	xla-miR-15c	xla-miR-429	xla-miR-19b		
NC_030730.1:112956827–112961455+	xla-miR-20	xla-miR-18	xla-miR-427	xla-miR-92a	xla-miR-23a
NW_016694899.1:44366-53978+	xla-miR-205				
NC_030728.1:76506768–76582180+	xla-miR-428	xla-miR-427	xla-miR-223	xla-miR-1b	
NC_030738.1:15457803–15459103+	xla-miR-1b				
NC_030739.1:94955628–94960533-	xla-miR-20	xla-miR-15c	xla-miR-18		
NC_030733.1:135562111–135567376+	xla-miR-1306				
NC_030736.1:34659166–34659570+	xla-miR-24b				
NC_030734.1:64782892–64788817-	xla-miR-427	xla-miR-428	xla-miR-223		
NC_030734.1:120248478–120250470+	xla-miR-18				
NC_030738.1:39776310–39778799-	xla-miR-1b	xla-miR-194	xla-miR-223	xla-miR-133a	xla-miR-133d
circRNA	miRNA	miRNA	miRNA	miRNA	miRNA
NC_030730.1:51360379–51374736+	xla-miR-427	xla-miR-18	xla-miR-24b	xla-miR-15c	xla-miR-428
NC_030740.1:46992144–46992645-	xla-miR-1306	xla-miR-1b			
NC_030725.1:11611649–11617677+	xla-miR-15c	xla-miR-1b	xla-miR-18	xla-miR-428	xla-miR-20
NC_030724.1:43830404–43835537+	xla-miR-15c	xla-miR-1b			
NC_030738.1:74755329–74775455+	xla-miR-205	xla-miR-429	xla-miR-15c	xla-miR-20	xla-miR-428
NC_030729.1:56464730–56475693-	xla-miR-428	xla-miR-427			
NC_030738.1:84049851–84051635-	xla-miR-15c	xla-miR-703	xla-miR-19b	xla-miR-23a	
NC_030736.1:16180713–16182409-	xla-miR-20				
NC_030724.1:211185447–211186783-	xla-miR-1306	xla-miR-18			
NC_030734.1:51792811–51831507-	xla-miR-18	xla-miR-429	xla-miR-15c		
NC_030734.1:57246068–57252093+	xla-miR-703				
NC_030738.1:88279403–88282053-	xla-miR-427	xla-miR-24b			
NC_030728.1:61473030–61481424-	xla-miR-20				
NW_016694908.1:92927-96080-	xla-miR-427	xla-miR-428	xla-miR-15c		
NC_030734.1:50722600–50728393+	xla-miR-92a	xla-miR-428	xla-miR-427	xla-miR-223	
NC_030733.1:17916295–17924623+	xla-miR-24b				
NC_030736.1:79090419–79101144+	xla-miR-20	xla-miR-427	xla-miR-428	xla-miR-18	
NC_030736.1:56496358–56501284+	xla-miR-15c	xla-miR-133d	xla-miR-133a	xla-miR-133b	
NC_030727.1:121417610–121420694+	xla-miR-205				
NC_030741.1:26301283–26301893-	xla-miR-427	xla-miR-23a			
NC_030735.1:32206764–32213523+	xla-miR-205				
NC_030739.1:77943686–77949468-	xla-miR-20	xla-miR-24b			
NC_030737.1:75371414–75374264+	xla-miR-427	xla-miR-428	xla-miR-194	xla-miR-18	
NC_030735.1:14601186–14607078-	xla-miR-223	xla-miR-15c			
NC_030739.1:70343848–70346398+	xla-miR-1b	xla-miR-18			
NC_030726.1:85184232–85191077+	xla-miR-23a				
NC_030738.1:79443529–79452085-	xla-miR-429	xla-miR-703	xla-miR-18	xla-miR-19b	xla-miR-223
NC_030735.1:20207460–20231569-	xla-miR-133d	xla-miR-133a	xla-miR-133b	xla-miR-1b	xla-miR-92a
NC_030741.1:98450239–98451611-	xla-miR-205				
NC_030729.1:21099855–21102101+	xla-miR-1b	xla-miR-429			
NC_030733.1:106477757–106479687+	xla-miR-205				
NC_030740.1:44305313–44305491-	xla-miR-427	xla-miR-20	xla-miR-428		
NC_030726.1:177942280–177943562-	xla-miR-427				
NC_030727.1:7116105–7120556+	xla-miR-427	xla-miR-19b	xla-miR-223	xla-miR-205	xla-miR-428
NC_030726.1:54224212–54254306-	xla-miR-20	xla-miR-92a	xla-miR-15c	xla-miR-363	xla-miR-427
NC_030739.1:83804071–83806502-	xla-miR-427	xla-miR-428			
NC_030734.1:48548571–48568404-	xla-miR-205	xla-miR-15c	xla-miR-1b	xla-miR-194	
NC_030740.1:2067198–2069340-	xla-miR-205				
NC_030725.1:8987622–8997647+	xla-miR-429	xla-miR-1306			
NC_030734.1:125970779–125973748+	xla-miR-24b	xla-miR-15c			
NC_030735.1:58608320–58651287-	xla-miR-429	xla-miR-92a	xla-miR-1b		
circRNA	miRNA	miRNA	miRNA	miRNA	miRNA
NC_030740.1:80604396–80624781+	xla-miR-92a	xla-miR-1b	xla-miR-142		
NW_016694822.1:2716268-2716475-	xla-miR-19b	xla-miR-133d	xla-miR-133a	xla-miR-133b	
NC_030738.1:38290681–38293041-	xla-miR-133a	xla-miR-223	xla-miR-133d	xla-miR-133b	xla-miR-142
NC_030731.1:52030005–52047024+	xla-miR-427	xla-miR-428	xla-miR-20	xla-miR-205	xla-miR-1b
NC_030725.1:117438012–117441967+	xla-miR-427	xla-miR-1b	xla-miR-15c	xla-miR-428	xla-miR-223
NC_030740.1:73055418–73056718+	xla-miR-24b	xla-miR-205			
NC_030737.1:31865134–31952348-	xla-miR-20	xla-miR-142	xla-miR-427	xla-miR-18	xla-miR-15c
NC_030732.1:17917996–17922482+	xla-miR-703				
NC_030737.1:47620341–47648895-	xla-miR-20	xla-miR-205			
NC_030733.1:39169680–39175656+	xla-miR-205				
NC_030724.1:104426383–104459491+	xla-miR-194	xla-miR-1b			
NC_030728.1:103186982–103187819+	xla-miR-142	xla-miR-20			
NC_030734.1:14617230–14633443-	xla-miR-427	xla-miR-363			
NC_030729.1:52242029–52243410-	xla-miR-427	xla-miR-20			
NW_016694810.1:2911866-2917505-	xla-miR-20	xla-miR-427	xla-miR-428	xla-miR-15c	xla-miR-18
NC_030726.1:29799927–29804271+	xla-miR-363	xla-miR-223	xla-miR-205		
NC_030733.1:68560883–68632668-	xla-miR-18	xla-miR-20	xla-miR-363	xla-miR-15c	xla-miR-427
NC_030733.1:35161214–35169222-	xla-miR-429				
NC_030726.1:137849524–137865898-	xla-miR-15c	xla-miR-92a	xla-miR-23a		
NC_030726.1:120212181–120225907-	xla-miR-15c	xla-miR-1b			
NC_030727.1:108870339–108903815+	xla-miR-429	xla-miR-23a	xla-miR-24b	xla-miR-703	xla-miR-19b
NC_030727.1:90727016–90750200+	xla-miR-428	xla-miR-427	xla-miR-194	xla-miR-20	xla-miR-15c
NC_030739.1:78251616–78253656+	xla-miR-133d	xla-miR-133a	xla-miR-133b		
NC_030740.1:26168837–26175753+	xla-miR-23a				
NC_030736.1:71193885–71201216+	xla-miR-427	xla-miR-20	xla-miR-428	xla-miR-205	xla-miR-23a
NC_030729.1:47521021–47526563+	xla-miR-427	xla-miR-428			
NC_030734.1:102704770–102705434+	xla-miR-427	xla-miR-23a	xla-miR-428		
NC_030734.1:19406998–19413599+	xla-miR-19b	xla-miR-223	xla-miR-15c	xla-miR-703	
NW_016694872.1:550852-632532-	xla-miR-18	xla-miR-363	xla-miR-20	xla-miR-1b	xla-miR-15c
NC_030731.1:77335237–77338872-	xla-miR-24b				
NC_030725.1:146024662–146027824+	xla-miR-429	xla-miR-133d	xla-miR-133b	xla-miR-133a	
NC_030730.1:67190695–67207690+	xla-miR-15c	xla-miR-703			
NC_030736.1:36038622–36040030+	xla-miR-363				
NC_030739.1:5643839–5666538+	xla-miR-205	xla-miR-18	xla-miR-24b		
NC_030732.1:108516037–108521820+	xla-miR-429	xla-miR-18	xla-miR-23a		
NC_030729.1:75130409–75139259-	xla-miR-223	xla-miR-1b	xla-miR-429	xla-miR-23a	xla-miR-205
NC_030724.1:132183425–132186548+	xla-miR-15c	xla-miR-427	xla-miR-428		
NC_030739.1:97616880–97621201+	xla-miR-427				
NC_030726.1:162054139–162061530-	xla-miR-133a	xla-miR-133b	xla-miR-133d	xla-miR-19b	
NC_030730.1:15778582–15786224+	xla-miR-23a	xla-miR-133d	xla-miR-133b	xla-miR-133a	xla-miR-363
NC_030735.1:52049437–52059905-	xla-miR-15c	xla-miR-142			
circRNA	miRNA	miRNA	miRNA	miRNA	miRNA
NC_030725.1:109818399–109821042-	xla-miR-23a				
NC_030725.1:97240333–97250262+	xla-miR-133d	xla-miR-133a	xla-miR-133b		
NC_030724.1:156827563–156854793-	xla-miR-205	xla-miR-92a	xla-miR-15c		
NC_030728.1:130323191–130324520-	xla-miR-24b	xla-miR-194			
NC_030731.1:115219575–115229231+	xla-miR-15c	xla-miR-194	xla-miR-18	xla-miR-703	xla-miR-23a
NC_030741.1:76210374–76210613-	xla-miR-18	xla-miR-15c			
NC_030727.1:107177485–107182142-	xla-miR-363				
NC_030728.1:95458531–95464536+	xla-miR-23a	xla-miR-133a	xla-miR-133d	xla-miR-133b	xla-miR-429
NC_030737.1:72301049–72303697+	xla-miR-363				
NC_030733.1:108871513–108873513-	xla-miR-15c				
NC_030740.1:26948790–26979571+	xla-miR-15c	xla-miR-194			
NC_030738.1:21126033–21131815+	xla-miR-19b	xla-miR-133d	xla-miR-133a	xla-miR-18	xla-miR-363
NC_030737.1:4840276–4844107-	xla-miR-703	xla-miR-15c			
NC_030730.1:86419246–86419592-	xla-miR-15c	xla-miR-428			
NC_030737.1:18147566–18150411-	xla-miR-363	xla-miR-133d	xla-miR-133a	xla-miR-133b	xla-miR-142
NC_030728.1:15545322–15553229+	xla-miR-1b	xla-miR-205			
NC_030734.1:87183400–87183864+	xla-miR-1306				
NC_030724.1:170644099–170647782+	xla-miR-427	xla-miR-428	xla-miR-205	xla-miR-19b	
NC_030732.1:52962177–52964782-	xla-miR-18				
NC_030738.1:104708016–104708272-	xla-miR-23a				
NC_030739.1:67484971–67488099-	xla-miR-427	xla-miR-15c	xla-miR-428	xla-miR-429	xla-miR-194
NC_030726.1:10872555–10886282-	xla-miR-223				
NC_030724.1:117241997–117249358-	xla-miR-18				
NC_030727.1:78958172–78962369+	xla-miR-15c	xla-miR-142	xla-miR-1b	xla-miR-19b	xla-miR-427
NC_030727.1:18870001–18886389-	xla-miR-133a	xla-miR-133d	xla-miR-133b	xla-miR-15c	xla-miR-427
NC_030733.1:135732775–135754096+	xla-miR-15c	xla-miR-133a	xla-miR-133b	xla-miR-133d	xla-miR-429
NC_030727.1:19553660–19564667-	xla-miR-18	xla-miR-20			
NW_016694812.1:6029247-6039595+	xla-miR-703				
NC_030726.1:153409169–153409658-	xla-miR-92a				
NC_030727.1:3769521–3772178-	xla-miR-142	xla-miR-19b	xla-miR-15c		
NC_030734.1:67440735–67446103-	xla-miR-427	xla-miR-428	xla-miR-23a	xla-miR-18	xla-miR-24b
NC_030734.1:149012368–149016101+	xla-miR-1b				
NC_030725.1:12051270–12078805+	xla-miR-15c				
NC_030726.1:166923803–166925161+	xla-miR-1b	xla-miR-363	xla-miR-1306		
NC_030741.1:271408–272630-	xla-miR-205				
NC_030730.1:17790904–17795162+	xla-miR-1b	xla-miR-205			
NC_030732.1:150988305–150989939+	xla-miR-1b				
NC_030727.1:18890380–18893603-	xla-miR-427	xla-miR-19b	xla-miR-428	xla-miR-15c	xla-miR-20
NC_030740.1:27551656–27584622+	xla-miR-20	xla-miR-142	xla-miR-428	xla-miR-1b	xla-miR-427
NC_030741.1:9140397–9144563+	xla-miR-18	xla-miR-428	xla-miR-20	xla-miR-19b	xla-miR-1b
NC_030740.1:85534431–85552303+	xla-miR-18	xla-miR-205	xla-miR-19b	xla-miR-194	
circRNA	miRNA	miRNA	miRNA	miRNA	miRNA
NC_030724.1:17377459–17378170-	xla-miR-92a				
NC_030740.1:36131571–36138031+	xla-miR-428	xla-miR-18	xla-miR-223	xla-miR-20	
NC_030741.1:101502504–101504659+	xla-miR-15c				
NC_030740.1:64855970–64900296+	xla-miR-194	xla-miR-205	xla-miR-19b	xla-miR-24b	xla-miR-18
NC_030733.1:2015378–2022047-	xla-miR-223	xla-miR-205			
NC_030734.1:110966435–110974339-	xla-miR-1b				
NC_030734.1:91534606–91543234-	xla-miR-15c	xla-miR-20	xla-miR-363	xla-miR-194	xla-miR-18
NC_030737.1:30055193–30055397-	xla-miR-429				
NC_030731.1:77496813–77500806-	xla-miR-427				
NC_030735.1:34874500–34913276-	xla-miR-15c	xla-miR-427	xla-miR-20	xla-miR-18	xla-miR-133a
NC_030728.1:55711908–55719138-	xla-miR-15c	xla-miR-1b			
NC_030730.1:87340543–87345501-	xla-miR-427	xla-miR-428			
NC_030728.1:103167247–103175214+	xla-miR-23a				
NC_030730.1:38529852–38532217-	xla-miR-23a	xla-miR-205	xla-miR-1b		
NC_030736.1:79090419–79096019+	xla-miR-427	xla-miR-428	xla-miR-20		
NC_030736.1:90287438–90296330-	xla-miR-19b	xla-miR-15c	xla-miR-92a	xla-miR-133a	xla-miR-20
NC_030730.1:8539270–8539599-	xla-miR-205				
NC_030725.1:82906739–82912357+	xla-miR-223	xla-miR-703	xla-miR-20	xla-miR-194	xla-miR-92a
NC_030740.1:111007219–111015557-	xla-miR-205	xla-miR-427			
NC_030724.1:143889932–143891419+	xla-miR-15c	xla-miR-427			
NC_030740.1:70765471–70766361-	xla-miR-1b	xla-miR-205	xla-miR-1306		
NC_030727.1:28031557–28054001-	xla-miR-205				
NC_030738.1:21875834–21876319+	xla-miR-15c				
NC_030731.1:70524254–70525545-	xla-miR-205				
NC_030729.1:48857151–48875366-	xla-miR-142				
NC_030729.1:95566328–95569412-	xla-miR-23a	xla-miR-1b	xla-miR-20	xla-miR-92a	xla-miR-427
NC_030727.1:60094099–60097903-	xla-miR-429	xla-miR-223	xla-miR-194		
NC_030736.1:77709534–77736150+	xla-miR-23a	xla-miR-18	xla-miR-205	xla-miR-15c	
NC_030740.1:85858236–85862849+	xla-miR-15c	xla-miR-427	xla-miR-142	xla-miR-1b	xla-miR-133a
NW_016694815.1:4530866-4536613+	xla-miR-133a	xla-miR-133b	xla-miR-133d		
NC_030725.1:127078532–127083938+	xla-miR-15c				
NC_030724.1:210401341–210403176-	xla-miR-15c	xla-miR-427	xla-miR-142	xla-miR-428	
NC_030725.1:163811669–163814745+	xla-miR-15c				
NC_030734.1:46013983–46031765-	xla-miR-23a	xla-miR-1b			
NC_030726.1:156345869–156347774-	xla-miR-18				
NC_030734.1:113723725–113729935+	xla-miR-92a	xla-miR-19b	xla-miR-15c	xla-miR-205	xla-miR-1b

## 2 Experimental design, materials and methods

### 2.1 Validation of differentially expressed circRNAs

We validated the expression of circRNAs identified by RNA-seq using Quantitative RT-PCR (Q-RT-PCR,ViiA 7 Real-time PCR System, Applied Biosystems, Carlsbad, CA, USA) for which we selected 8 circRNAs with the divergent primers as listed in Table 1. The circRNAs were chosen based on their functional roles and respective FC values. For Q-RT-PCR analysis, total RNA was converted into cDNA using the Invitrogen Superscript cDNA Synthesis kit (Invitrogen Corp., Carlsbad, CA, USA). Reactions were performed according to the manufacturer's instructions. Relative circRNAs expression was calculated according to the 2-△△CT method [6]. The upregulated circRNAs were identified at 2-△△CT >1, the downregulated circRNAs were identified at 2-△△CT<1. The expression of gene gpi.S (NM_001092296.1) was used as a reference for data normalization, because it was reported to be a housekeeping gene [7]. The Q-RT-PCR assays were performed in three replicates. Statistical analysis was conducted using the SPSS Statistics 18.0 software (IBM Corp., New York, NY, USA). Significant difference between control and AZ-treated groups was compared using student's *t*-test at *p*< 0.05.

### 2.2 Bioinformatic analysis

KEGG analysis were performed for the differentially expressed circRNA-associated target genes [5], [8]. MiRNA-binding sites on circRNAs are all predicted by custom-written software based on Targetscan and Miranda software (Cloud-Seq Biotech Ltd. Co., Shanghai, China).
