# Supplementary material for: Validation and bioinformatics analysis of differentially expressed circRNAs involved in developing male Xenopus laevis chronically exposed to atrazine
Source: Data Brief. 2018 Apr 10;18:1282–91. doi: 10.1016/j.dib.2018.04.011 (PMC5996731; doi:10.1016/j.dib.2018.04.011)
Supplement: Supplementary file 1 — Supplementary material. [file mmc1.doc]

**Conflict of Interest Form**

The authors declare no conflict of interest. The authors alone are responsible for the content and writing of the paper.
